# Structural characteristics of specialised living units for people with dementia: a cross-sectional study in German nursing homes

**DOI:** 10.1186/1752-4458-8-39

**Published:** 2014-10-21

**Authors:** Rebecca Palm, Sabine Bartholomeyczik, Martina Roes, Bernhard Holle

**Affiliations:** German Centre for Neurodegenerative Diseases, Stockumer Str. 12, 58453 Witten, Germany; Faculty of Health, School of Nursing Science, Witten/Herdecke University (UW/H), Stockumer Str. 12, 58453 Witten, Germany

**Keywords:** Dementia, Dementia special care unit, Small-scale living unit, Structural characteristics

## Abstract

**Background:**

Living units (LU) specialised for people with dementia are an important feature of nursing homes. Little is known about their structural characteristics, and an international definition is lacking. This study explored characteristics of the environment and staff from defined LU types to identify differences between them.

**Design:**

Cross-sectional study comparing five types of LUs. LUs were defined based on their living concept (segregated and integrated), size (small and large scale), and funding (extra funded and not extra funded). Differences were identified using descriptive statistics, Chi-Square resp. Kruskal-Wallis-Test and post-hoc analysis with Bonferroni corrections.

**Results:**

In total, 103 LUs from 51 nursing homes participated: 63 integrated and 40 segregated LUs; 48 integrated and 31 segregated LUs were large. Sixteen large segregated LUs were extra funded. Regarding the environment, a distinctive feature of small LUs was a higher percentage of single rooms. Small integrated LUs provided and served meals more in a homelike manner than other LUs. LUs did not differ in their interior and access for the residents to use outdoor areas. Regarding the staff, small LUs provided more staff, but they were not exclusively assigned to the LUs. Large segregated LUs with additional funding provided more registered nurses and nurses with a special qualification per resident than the other large LUs.

**Conclusion:**

Nursing homes implemented different features in their specialised LUs. Because single room availability, homelike provision of meals, staff quantity, quality and continuity may influence residents’ outcomes, it is necessary to investigate whether differences are apparent in future evaluation studies of specialised LUs for people with dementia.

## Background

The number of people with dementia (PwD) is increasing worldwide [1]. The majority live at home and wish to stay there as long as possible. However, with the progression of cognitive decline, behavioural and functional restrictions become more demanding so that a considerable amount of people with dementia move into a nursing home [2]. In many high-income countries, nursing home care is a significant feature of long-term care that is supported by the country in the form of funding and resources [3].

In the past few decades, nursing homes have adapted their concepts to the special needs of PwD; one method is specialised living units (LU) for PwD. The implementation of specialised LUs is a subject of national dementia strategies [4–6] and is promoted by national Alzheimer Societies [7] and Alzheimer’s International [1]. In Germany, the implementation of specialised LUs for PwD is recommended by researchers in the national guideline for people with challenging behaviour in nursing homes [8] and the Medical Advisory Services of the statutory health insurance [9]. Because of the increasing number of PwD in nursing homes and the preference for specialised nursing home care, specialised LUs for PwD are mushrooming worldwide [10].

Various types of specialised LUs for PwD with a heterogeneous structure were developed worldwide. They differ regarding their philosophy, environmental design, therapeutic approach [11]. Until now, an international consensus on a definition of the various types of specialised LUs for PwD has not existed. In Germany, several types were developed and implemented in ca. 30% of the nursing homes [12]. Like in other countries, a national definition on the special features of these units is missing, therefore it is not known when a LU labels itself “specialised for PwD”, which concepts are associated with this label and what characterises the concept of the LU. However, the size of a LU and the living concept appear to be integral aspects of the concepts [13–15].

In the literature we identified two main types of specialised LUs for PwD:

1. Large segregated LUs (Dementia Special Care Units, [Spezielle Dementenbetreuung]): hosts in general more than fifteen residents diagnosed with dementia who exhibit severe challenging behaviour; residents are dependent in daily functioning and are able to move independently or with little help. Inclusion criteria regulate the admission to the LU.
2. Small segregated LUs (small-scale homelike LUs, group living, [Haus- und Wohngemeinschaft]): a group living concept for five to fifteen residents with dementia who are mobile. Inclusion criteria are not obligate, but homogenous groups with regard to the severity of cognitive impairment and challenging behaviour are persuaded.

Large segregated LUs focus on the impairment of PwD. They aim to compensate memory-loss, disorientation and challenging behaviour by adapting the environment, care features and offer special programs conceptualised for PwD (e.g. memory training, reminiscence therapy, validation therapy). The staff is specialised by on-going training and support, and the LUs have a designated leader who is highly educated [10, 16–18]. The other concept - small scale LUs - is also conceptualised for PwD but differ from large segregated LUs. The concept of small scale LUs emphasises the beneficial effects of a homelike environment and the normalisation of daily life [19–24]. Instead of offering special therapeutic programs residents are involved in domestic tasks and occupational group activities. In contrast to large segregated LUs, less nurses but more nursing assistants and service staff are employed. Continuity of staff is enhanced by the integration of nursing and domestic tasks [19].

In Germany, also small scale LUs with integrated living concepts exists. They address mainly residents with dementia, but residents without a diagnosis or cognitive impairments also live in these LUs. Another important criterion, which distinguishes LUs, is their funding. With additional funding LUs have the possibility to improve structural conditions, such as staff ratios and qualification. In Germany, the provision of special care is the rationale for service providers to charge higher rates to reimburse costs. The rates are paid by the resident or by the social welfare agency if the resident can't pay for himself. The amount of these rates is negotiated by representatives of the statutory long term care insurance agencies and the municipalities. Because the additional costs should only be allocated for special services in dementia care, the adherence is controlled by the Medical Service of Health Funds. In three of the sixteen federal states (Hamburg, Berlin, Baden-Württemberg), the criteria for specialised LUs and their costs are defined in legal agreements on the state level; in other federal states, they are negotiated.

Despite the high number of nursing homes with any type of specialised LUs for PwD, the majority of residents with dementia are living in so called “traditional” LUs, which hosts in general more than 20 residents with or without dementia.

Because of the importance of determining what nursing home setting best serves PwD, a recent systematic review was conducted [25]. It reviewed the evidence of structural characteristics, such as material resources (e.g., single rooms, familiar homelike components, access to outdoors), human resources (e.g., level of staffing, expertise of staff) and their operation (e.g., nurse-resident ratios, consistency of assignment). The authors found sparse evidence from non-experimental trials demonstrating the benefit of setting-related characteristics because the diversity of the studied variables exacerbated the pooling of data. As a result, no strong evidence of combined findings can facilitate decision-making. Currently, we are facing several difficulties: evaluating the effects of certain LU types; interpreting measured quality outcomes; deciding on the regulation of these units and subsequently choosing the most suitable offer.

To conduct future evaluation studies of specialised LUs, Zimmerman et al. [25] suggested examining differences between nursing home settings to identify key variables that are related to outcomes of PwD. Sloane et al. [26] postulated nearly 20 years ago four goals to develop the methods needed to conduct studies on the effectiveness of specialised LUs for PwD: the description of LUs and their occupants, the improvement of basic definitions, the creation of typologies of LU care modalities and the identification of the variability amongst LUs. Particularly in Europe, these goals have not been reached.

The longitudinal study DemenzMonitor aims to investigate psychosocial outcomes of residents with dementia from various LU types in nursing homes [27]. Because a national and international accepted definition of dementia-specific LUs and their structural characteristics is lacking, we acted on the suggestion of Zimmerman et al. [25] and Sloane et al. [26] and aimed to explore the differences in structural characteristics of various LU types in German nursing homes in this article. LUs were defined based on their living concept (segregated and integrated), size (small and large scale), and funding (extra funded and not extra funded). The comparison focuses on LU characteristics of the environment (unit size, architectural features, features of the interior and outdoor area, and features of homelike components) and staff (resident-staff ratios, qualification of the head nurse, staff assignment).

Based on the theoretical concepts of the various LUs, large segregated LUs should provide more specialised staff than others; small scale LUs offer a more homelike environment and have more non-specialised staff. However, we assumed that differences may not appear clearly because definition criteria are missing and features of dementia-specialised LUs may also have been implemented by non-specialised LUs [28].

## Methods

### Design and sample

Cross-sectional data from a convenience sample of 103 LUs in 51 nursing homes were used. The data were derived from the 2013 measurement cycle of the DemenzMonitor study [27]. Nursing homes that were eligible to participate were defined according to the German statutory long term care insurance law: they are hosting persons who exhibit a care dependency and receive reimbursement through the statutory long-term care insurance. Representatives of the Medical Service of Health Funds assess nationwide dependency on care, using a standardized instrument. This allows a categorisation in three care levels (care level 1-considerable care dependency/2- severe care dependency/3- very severe care dependency). Care assessments are usually performed prior to nursing home admission but can be updated if changes occur.

There were no in- or exclusion criteria for nursing homes to participate; a variety was intended. The participating sample was not selected; nursing homes that declared their interest to participate were enclosed. All nursing homes participated voluntarily.

### Data collection

The data were assessed in May 2013 by the nursing home staff using a standardised questionnaire. At the time point of data collection, no validated questionnaire for the assessment of environmental characteristics that additionally could be completed by the nursing home staff was available - either as a translated version of existing tools or with its origin in Germany. Therefore, a questionnaire was developed and tested by experts and future users. The content validity and practicality of the questionnaire was evaluated using expert interviews and a multi-method pre-test. Problematic items were adapted and tested again until an acceptable data quality could be guaranteed.

Data were assessed on the level of the nursing home, the LU and the resident in three separate questionnaires. In each nursing home, a staff member was designated as a study coordinator and was responsible for the entire data collection process. The study coordinator was prepared by a one-day training given by the research team. During the training, each item on the questionnaires was explained and examples were given. Additionally, an item handbook was provided were each item was defined. The LU questionnaire was usually completed by the head nurse; the nursing home questionnaire was completed by the nursing home manager; the residents’ questionnaire was completed by a registered nurse familiar with the resident. The study coordinator was the initial contact person for questions of the assessors and supervised the completion of the questionnaire. The research team could also be contacted to answer questions.

After completion, the data sets were checked for completeness and plausibility. To correct or replace missing or implausible items, either the study coordinator was contacted or data of stable items were imputed from the previous measurement cycle. Data that could not be replaced were not analysed.

Further details on the data collection are described elsewhere [27].

### Definition and measures

Since a definition of the different types of specialised LUs is missing, three structural criteria were used to define the LUs:

1. Living concept: integration (residents with and without dementia are living together in a LU)/segregation (exclusively residents with dementia are living in the LU)
2. Size: small (≤15 residents)/large (>15 residents)
3. Funding: extra funded/not extra funded

Based on these criteria, eight groups of different LUs were possible. However, in the data we found five groups because none of the small LUs and large integrated LUs received extra funding.

- ▪ Large segregated living units (LSLU) with extra funding
- ▪ Large segregated living units (LSLU) without extra funding
- ▪ Small segregated living units (SSLU) without extra funding
- ▪ Large integrated living units (LILU) without extra funding
- ▪ Small integrated living units (SILU) without extra funding

We operationalised items that were described as structural features of specialised LUs in the literature [25]. The items comprised environmental characteristics (LU size, type of room, architectural features, and features of the interior and outdoor area). As characteristics of a homelike atmosphere, we chose two items that describe meal provision (lunch is cooked in the LU, meal serving system). Staff provision was measured as staff organisation, qualification and resident-to-staff ratios. An overview of the assessed structural characteristics is listed in Table 1.

**Table 1:** **Operationalisation of structural characteristics**

Variable	Description of the assessed item/calculation of the variable	Level
*Environmental characteristics*		
Size	Number of beds in the LU	1
Single rooms	Number of single rooms in the LU	1
Building specific for residents with dementia	The LU was built to host residents with dementia.	2
Architectural segregation from other units	The LU is located in a separate building or floor or segregated by a closed door.	2
Exit control	The living unit is protected by exit controls.	2
Permission to bring own furniture	Residents are allowed to bring own furniture.	2
Furnishing of public rooms		2
▪ is solely functional	Functional furniture is provided by the institution and designed for a special use.	
▪ functional and individual	Individual furniture is purchased from private people.	
▪ solely individual		
Accessible outdoor area	An accessible outdoor area is defined as a garden, terrace or balcony that can be entered directly from the LU. If residents can go out alone, it is designed without any barriers.	2
▪ residents can go out alone		
▪ residents can go out with attendance		
▪ no outdoor area		
Preparation of meals in the LU	Meals are prepared in the kitchen of the LU.	2
Cooking lunch	Lunch is cooked in the kitchen of the LU.	2
Meal serving system	^A^Meal serving system for the majority of the residents	2
▪ Tray system	^A^All meals are already prepared on a tray when they arrive on the LU.	2
▪ Dish system	^A^All meals are portioned out by the staff individually for each resident on a plate.	
▪ Buffet system	^A^All meals are self-service on a buffet.	
▪ Homelike table system	^A^All meals are served homelike on the table.	
▪ Mixed system	Different systems are used for breakfast, lunch and dinner.	
*Staff ratios*		
Residents-per-registered nurse	Number of residents on the day of data collection/number of registered nurses (defined as nurses with a minimum education of three years)	1
Residents-per-certified nursing assistant	Number of residents on the day of data collection/number of certified nursing assistants (defined as nurses with a minimum education of one year)	1
Residents-per-nursing assistants	Number of residents on the day of data collection/number of nursing assistants (defined as nurses without any education)	1
Residents-per-service staff member	Number of residents on the day of data collection/number of service workers	1
Staff assignment and qualification		
▪ Constant assignment (nurses)	Nurses are working exclusively in one designated LU.	2
▪ Constant assignment (service staff)	Service workers are working exclusively in one designated LU.	2
▪ Continuous presence of a registered nurse	A registered nurse is always present during day shift in the LU.	2
▪ Special qualification of head nurse in psychogeriatric care	The head nurse of the LU has a special qualification in psychogeriatric care.	2

^A^All meals include breakfast, lunch and dinner.

1 Numeric; 2 Dichotomous.

To calculate the staff ratios, head nurses were asked to specify the number of staff (counted in persons) that were present in four defined time periods during early and late shift. We analysed all four time periods, but report only one (early shift 08.00-09.00 AM) because the staff ratios were similar. We are aware that a more comprehensive method to illustrate staff provision is the calculation of staff hours per day per resident. Because the number of staff can’t be derived automatically in the institutions but staff has to calculate the numbers, we decided to use defined short time periods to calculate ratios because the assessment is faster and more fail-safe.

### Statistical analysis

We calculated absolute and relative frequencies for dichotomous variables and distribution parameters of numeric variables to describe unit characteristics. Differen-ces between frequency distributions of dichotomous variables were tested for significance using *χ*^*2*^ test.

To test differences between the means of the various groups, assumptions for variance analysis were checked (Kolmogorov-Smirnov Test for normal distribution; Brown-Forsythe Test for Equality of Means). Data were found to be normally distributed, but the variance was not homogenous. In the case of unequal group sizes, the violation of any assumption may cause invalid results of variance analysis. Therefore, a nonparametric test (Kruskal-Wallis Test) was performed to test differences between the groups. Mann–Whitney tests with Bonferroni corrections were performed as post-hoc procedures to follow up significant results from the Kruskal-Wallis-Test and to compare groups. The descriptive results indicated that it was not reasonable to test each group with each other but to test only select groups with a Mann–Whitney-Test. Because the differences between large and small LUs were obvious, we tested group differences only within large and small LUs. To avoid Type I error in comparing the three groups of large LUs, the level of significance for comparisons between more than two groups was adjusted according to Bonferroni (α = .005/3 = .0167). The significant results of the post-hoc analysis were reported. Test procedures were conducted using SPSS Statistics 21.

A sample size calculation was not performed because of the explorative nature of the study. Significance values were not interpreted to reject a hypothesis but to identify relevant differences.

### Ethical considerations

The ethics committee of the German Society of Nursing Science approved the study. In the ethical evaluation, special attention was given to data security. The security was guaranteed by collecting the data under a pseudonym and storing the data according to the German national privacy policy.

## Results

### Participants

103 LUs participated and returned the questionnaire. The participating LUs were located in 51 nursing homes from 11 German federal states. 70% (36/51) nursing homes were located in an urban area (>20.000 inhabitants), and 66% (34/51) were run by a non-profit organisation. There was large variance in the size of the nursing homes (mean 124 ± 64.61, min 26, max 250).

**Figure 1:**
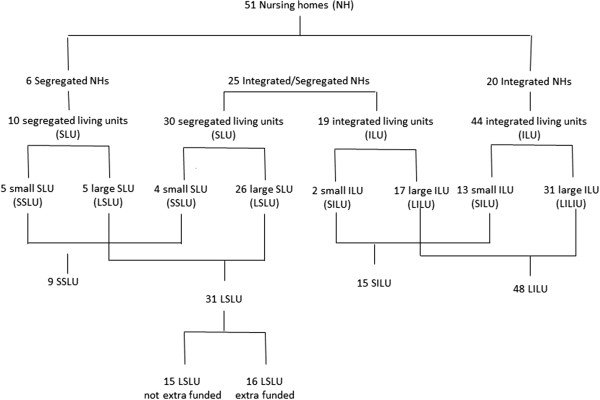
**Sample flow chart of living unit clusters.**

The majority of the nursing homes were specialised for residents with dementia: 12% (6/51) were specialised nursing homes exclusively for residents with dementia, and 45% (23/51) offered segregated LUs for residents with dementia. 43% (22/51) had integrated LUs. The number of participating LUs from each nursing home differed: on average, two LUs from each nursing home participated, with a minimum of one and a maximum of six LUs per nursing home. This caused a clustering of LUs from one nursing home in some of the groups. The classification according to the defined criteria of the LUs and number of participants is shown in Figure 1.

In the 103 LUs 2481 residents were living in the LU, 1808 were assessed in the data collection. Residents’ characteristics are shown in Table 2.

**Table 2:** **Residents characteristics of various LUs**

	LSLU I	LSLU II	LILU	SSLU	SILU
Total n	285	274	994	99	154
Age (mean, years) ± SD (Min-Max)	83.2 ± 7.5 (59–101)	80.8 ± 8.9 (48–99)	83.5 ± 9.1 (44–106)	82.7 ± 9.1 (46–96)	83.4 ± 9.0 (46–99)
Sex (female), n (%)	221 (77.5)	203 (74.1)	754 (75.8)	72 (72.7)	116 (75.3)
Care level					
No care level	0	1 (0.4)	15 (1.5)	1 (1.0)	2 (1.3)
1 (considerable care dependency)	59 (20.7)	46 (16.8)	328 (33.0)	26 (26.3)	63 (40.9)
2 (severe care dependency)	146 (51.2)	133 (48.5)	425 (42.8)	38 (38.4)	68 (44.2)
3 (very severe care dependency)	80 (28.1)	94 (34.3)	226 (22.7)	34 (34.3)	21 (13.6)
Diagnosis of dementia, n (%)	258 (90.5)	270 (98.5)	611 (61.5)	88 (88.9)	100 (64.9)
Dementia Severity^1^					
No dementia, n (%)	22 (7.7)	2 (0.7)	281 (28.4)	7 (7.1)	50 (32.5)
Mild-moderate dementia, n (%)	60 (21.1)	33 (12.0)	339 (34.2)	25 (25.3)	46 (29.9)
Severe dementia, n (%)	203 (71.2)	239 (87.3)	371 (37.4)	67 (67.7)	58 (37.7)

LSLU I-Large Segregated Living Unit not extra funded; LSLU II- Large Segregated Unit extra funded;

SSLU-Small Segregated Living Unit; LILU-Large Integrated Living Unit; SILU-Small Integrated Living Unit.

SGB XI – Social insurance law XI.

^1^assessed with the Dementia Severity Scale [29].

### Environmental characteristics

Particular characteristics of the architecture differed significantly between the groups. Large LUs had significantly more beds than small LUs (H (4) = 57.26, p < .001), but no significant difference was found within the groups of small and large LUs. Both types of small LUs were providing significantly more single rooms than large LUs. Large integrated and segregated LUs without additional funding had a comparable room structure; large segregated LUs with additional funding were providing the fewest single rooms.

Of all three types of segregated LUs, 87% (35/40) were built/rebuilt for residents with dementia. The highest percentage was found in the group of large segregated LUs with extra funding (93%). One feature of LSLU with extra funding was exit control, which existed in 50% (8/16) of these units but in 22% (9/40) of other segregated LUs. 81% (13/16) LSLUs with extra funding were in a separate building or separated from other units by other architectural conditions, compared to 73% (11/15) of LSLUs without extra funding and 55% (5/9) of SSLUs. However, 93% (14/15) of small integrated LUs were also in a separate building.

There were no differences found between the groups regarding their interior and outdoor areas. In nearly all LUs, residents could bring their own furniture, and the majority of all LU public rooms were furnished with a mix of functional and individual furniture. The majority of LUs provided an outdoor area that the residents could use without supervision.

Meals were provided differently in integrated and segregated LUs. In 75% (78/103) LUs, head nurses indicated that meals were prepared in the LU; we found no differences between the groups. In 28 LUs, this meal preparation involved cooking lunch. The percentage of LUs that cook their lunch themselves was highest in small integrated LUs. In this study, 80% (12/15) indicated that lunch is cooked in the LU, whereas in the other groups, less than 50% did. Likewise, 60% (9/15) of small integrated LUs indicated that all meals were served homelike on the table, which only occurred in less than 30% of the other LUs.

The results of the environment characteristics are shown in Table 3.

**Table 3:** **Environmental characteristics of various LUs**

	LSLU I	LSLU II	LILU	SSLU	SILU		
Total n	15	16	48	9	15	Test statistics	P-value
*Architecture*							
Number of beds, median (range)	24.0 (16–63)	30.0 (21–40)	28.0 (17–55)	11.0 (9–15)	12.0 (5–14)	H (4) = 57.26	<.001
Percentage of single rooms, median (range)	78.8 (14.2-92.0)	50.0 (0–96.0)	83.3 (5.8-100.0)	100.0 (90.9-100)	100.0 (72.7.-100.0)	H (4) = 48.61	<.001
Built for residents with dementia, n (%) yes	12 (80.0)	15 (93.8)	7 (14.6)	8 (88.9)	5 (33.3)	*χ* ^*2*^ (4) = 48.42	<.001
Architectural separation from other units,n (%) yes	11 (73.3)	13 (81.3)	28 (58.3)	5 (55.6)	14 (93.3)	*χ* ^*2*^ (4) = 8.70	.006
Exit control, n (%) yes	3 (20.0)	8 (50.0)	4 (8.3)	1 (11.1)	1 (6.7)	*χ* ^*2*^ (4) = 16.72	.002
*Interior and outdoor area*							
Permission to bring own furniture, n (%) yes	14 (93.3)	16 (100)	48 (100)	9 (100)	15 (100)	*χ* ^*2*^ (4) = 5.92	.205
Furnishing of public rooms, n (%) yes							
Solely functional	3 (20.0)	2 (12.5)	6 (12.5)	2 (22.2)	0	*χ* ^*2*^ (8) = 6.00	.646^2^
Functional and individual	12 (80.0)	13 (81.3)	41 (85.4)	7 (77.8)	15 (100)		
Solely individual	0	1 (6.3)	1 (2.1)	0	0		
Accessible outdoor area, n (%) yes^1^							
Residents can go out alone	10 (66.7)	13 (81.3)	40 (85.1)	6 (66.7)	13 (86.7)	*χ* ^*2*^ (8) = 8.03	.431^2^
Residents can go out with attendance	3 (20.0)	3 (18.8)	5 (10.6)	1 (11.1)	1 (6.7)		
No outdoor area	2 (13.3)	0	2 (4.3)	2 (22.2)	1 (6.7)		
*Meal provision*							
Preparation of meals in the LU, n (%) yes	11 (73.3)	14 (87.5)	31 (64.5)	7 (77.8)	15 (100)	*χ* ^*2*^ (4) = 9.32	.053
Cooking lunch, n (%) yes^3^	5 (45.5)	2 (14.3)	6 (12.5)	3 (33.3)	12 (80.0)	*χ* ^*2*^ (4) = 19.79	.001
Meal serving system, n (%) yes							
Tray system	0	0	2 (4.2)	0	0	*χ* ^*2*^ (12) = 24.44	.018^2^
Dish system	2 (13.3)	7 (43.8)	16 (33.3)	3 (33.3)	2 (13.3)		
Buffett system	0	0	0	0	0		
Homelike table system	4 (26.7)	2 (12.5)	4 (8.3)	2 (22.2)	9 (60.0)		
Mixed system	9 (60.0)	7 (43.8)	26 (54.2)	4 (44.4)	4 (26.7)		

LSLU I-Large Segregated Living Unit not extra funded; LSLU II- Large Segregated Unit extra funded;

SSLU-Small Segregated Living Unit; LILU-Large Integrated Living Unit; SILU-Small Integrated Living Unit.

^1^The total n for this item is 47 because of one missing answer.

^2^Results of the χ^2^ test may be invalid because the frequency is zero in several cells.

^3^Reffering group are the LU where meals are prepared.

### Characteristics of staff organisation and qualification

All large LUs and 88% (8/9) of the small segregated LUs stated that nursing staff was constantly assigned to one LU. In small integrated LUs, 60% (9/15) indicated this assignment, which means that the allocation of nursing staff is more flexible. However, the amount of small integrated LUs that assign their service staff constantly is higher (93%; 14/15). This procedure does not occur in the other LUs, where the amount of constant allocated service staff is lower than nursing staff.

The continuous presence of a registered nurse during the day shift was a characteristic of large LUs but not consistent across all small LUs. All large segregated LUs and 95% (46/48) of the large integrated LUs stated that the continuous presence of a registered nurse during the day shift applied to their staff concept. In small LUs, the amount was lower (77% (7/9) in small segregated LUs and 60% (9/15) in small integrated LUs.

The head nurse was specialised in psychogeriatric nursing in 81% (13/16) of the large segregated LUs with additional funding. The amount of LUs with a head nurse with a special qualification was below 50% in the other groups.

Results are shown in Table 4.

**Table 4:** **Staff characteristics of various LUs**

	LSLU I	LSLU II	LILU	SSLU	SILU		
Total n	15	16	48	9	15	Test statistic	p-value
Constant assignment (nurses), n% yes	15 (100)	16 (100)	48 (100)	8 (88.9)	9 (60)	*χ* ^*2*^ (4) = 31.33	<.001
Constant assignment (service staff), n% yes	14 (93.3)	9 (56.3)	36 (75.0)	4 (44.4)	14 (93.3)	*χ* ^*2*^ (4) = 12.77	.012
Continuous presence of a registered nurse, n% yes	15 (100)	16 (100)	46 (95.8)	7 (77.8)	9 (60)	*χ* ^*2*^ (4) = 22.32	<.001
Special qualification of head nurse in psychogeriatric care, n% yes	2 (13.3)	13 (81.3)	4 (8.3)	4 (44.4)	3 (20)	χ^2^ (4) = 36.97	<.001

LSLU I-Large Segregated Living Unit not extra funded; LSLU II- Large Segregated Unit extra funded;

SSLU-Small Segregated Living Unit; LILU-Large Integrated Living Unit; SILU-Small Integrated Living Unit.

### Staff ratios

The median (mdn) of all staff ratios (except residents-per-certified nursing assistants) differed statistically significant between the five groups. They indicate that in large LUs, the staff has to care for twice as many residents than in small LUs. However, the results also indicate that in a higher percentage of small LUs, a registered nurse was not present, whereas a registered nurse was present in nearly all large LUs. The results of staff ratios are shown in Table 5.

**Table 5:** **Staff ratios of various LUs in early shift**

	LSLU I	LSLU II	LILU	SSLU	SILU	Test statistic	p-value
LUs with RNs, n	15	15	48	7	10		
LUs without RNs, n	0	0	0	2	5		
Residents-per-RN, median (range)	19.5 (8.2 - 52)	16.0 (7.7-28)	20.5 (7–50)	9.0 (5–15)	12.0 (5–14)	H (4) = 29.42	<.001^1^
LUs with CNAs, n	7	2	12	3	7		
LUs without CNAs, n	8	14	36	6	8		
Residents-per-CNA, median (range)	25.0 (12–52)	18.0 (15–21)	21.5 (15–31)	9.0 (7–10)	12.0 (10–14)	H (4) = 19.90	.001^2^
LUs with NAs	12	14	43	4	4		
LUs without NAs	3	2	5	5	11		
Residents-per-NA, median (range)	13.5 (10–26)	11.0 (8.5-31)	15.0 (5–40)	10.0 (7–13)	11.5 (5–12)	H (4) = 8.59	.075
LUs with SSMs	10	8	33	7	12		
LUs without SSMs	5	8	15	2	3		
Residents-per-SSM, median (range)	25.5 (16–56)	29.0 (16–56)	26.0 (8.6-40)	11.0 (5–14)	11.5 (10–14)	H (4) = 35.90	.001

LSLU I-Large Segregated Living Unit not extra funded; LSLU II- Large Segregated Unit extra funded;

SSLU-Small Segregated Living Unit; LILU-Large Integrated Living Unit; SILU-Small Integrated Living Unit.

RN–Registered nurse; CNA–Certified-nursing-assistant; NA–nursing assistant, SSM – service staff member.

^1^Post-hoc analysis revealed statistically significant differences between LSLU II and LILUs (U = 212.5, z = -2.66, p = .008, r = -0.33).

^2^Post-hoc analysis revealed statistically significant differences between SSLU and SILU (U = 1.00, z = -2.26, p = .024, r = 0.71).

Amongst the large LUs, we found the highest ratio of residents per registered nurses (RN) in integrated LUs (mdn 20.5); the lowest ratio was in segregated LUs with additional funding (mdn 16.0). The Mann–Whitney Test revealed that this difference was statistically significant at a α = .0167 level (U = 212.5, z = -2.66, p = .008, r = -0.33). Large LUs without additional funding did not differ statistically significant in the amount of registered nurses. The small LUs did also not differ in the ratio of residents-per-RN.

The ratios for residents-per-certified nursing assistants (CNA) were comparable to those of RNs amongst all groups. We found no statistically significant difference between the groups of large or small LUs. In the small LUs, the residents per CNA ratio was higher in integrated LUs at a α = .05 level (U = 1.00, z = -2.26, p = .024, r = 0.71). The results have to be interpreted cautiously, because they are based on a small sample of LUs where CNAs were present during the assessed time periods: in 58% (14/24) of small LUs and 73% (58/79) of large LUs, a CNA was not present in the assessed time period.

Nursing assistants (NA) were the only staff group in large and small LUs that were caring for a comparable number of staff per resident. For the small LUs, the results were based on a small sample, because 66% (16/24) of LUs did not provide NAs.

Regarding service staff, we found the same pattern as the resident-to-nursing staff ratios: the ratios were about twice as high in large LUs as in small LUs. There were no significant differences between the large LUs or between the small LUs during any shift.

## Discussion

This study aimed to compare structural characteristics of LUs specialised in caring for people with dementia and other non-specialised forms in German nursing homes to provide a basis for future evaluation studies. The definition of LU types was based on two central aspects that are described in the literature: living concept and size of the LU. Furthermore, we considered the influence of additional funding for LUs, which is a distinctive feature of the German long term care insurance system.

The results must be discussed against the background of the resident’s structure of the LUs. Segregated LUs hosted considerably more residents with dementia; residents from large specialised LUs showed more often symptoms of severe dementia.

Our results showed the following:

1. A building which was constructed for PwD is a distinctive feature of segregated but not of integrated LUs.

Integrated LUs are often not built or rebuilt for PwD, and therefore, they most likely are more similar to traditional nursing homes. Literature has stated that traditional nursing homes that were not particularly built for residents with dementia do not meet their needs [5]. A recent review summarised the evidence for the design of environments for PwD in long-term care [30]. They concluded that there is no clear effect of small and homelike environments; more recent studies also cannot confirm this finding [20, 21, 31]. A reason may be that it is unclear which design aspects of a homelike environment are distinctive for small scale LUs.

2. A higher percentage of single rooms differentiate large and small scale LUs.

Our results revealed that the provision of single rooms is a feature of small-scale LUs. Single rooms facilitate the resident’s privacy and create a more homelike atmosphere. Moreover, disruptions during the night can be reduced. These aspects are compromised in double or shared rooms. The review of Fleming and Purandare [30] emphasises the beneficial effects of single rooms on PwD in nursing homes, but it refers to only one study of moderate quality. In published small-scale evaluation studies [20, 21, 31], the room structure was neither considered in the eligibility criteria nor reported, so we do not know if this factor contributes to resident outcomes.

3. LUs do not differ with regard to their interior and outdoor area.

Because we did not find any differences regarding the characteristics of the interior and access to outdoor areas between the LUs, we concluded that traditional nursing homes implemented aspects of dementia-specific living concepts [28].

4. Small integrated LUs and large segregated LUs with additional funding are more often separated from other LUs in the nursing home than the other types of LUs.

The separation of special LUs from other LUs in the nursing home is often used as a criterion to define them [16, 21]. The definition of Maslow [16] also includes the control of entry in and egress from the LU, a criterion which we also found in the group of large segregated LUs with additional funding. The majority of small segregated LUs in our sample were not separated from the rest of the nursing homes. In Germany, these LUs are quite often operated as wings or special areas in large integrated LUs or nursing homes. If a definition of specialised LUs for PwD would include the separation of the LU from the rest of the nursing home, those wings and special areas would be excluded. Additional definitions of specialised LUs for PwD explicitly include wings or special areas [10]. In our opinion, the question if a LU is separated from the rest of the nursing home affects other organisational procedures (e.g., meal provision). In separated LUs, it may be easier to implement a different meal provision system, such as self-cooking.

5. Cooking lunch in the LU and a homelike meal serving system is a distinctive feature of small integrated LUs.

We explained above that the separation of the LU from the nursing home may enhance self-cooking in the LU, and therefore, LUs which are not separated may have more difficulty implementing this feature. Te Boekhorst et al. [32] showed that in small scale LUs, cooking dinner was practiced in the Netherlands but not in traditional LUs. In their study, small scale LUs were defined as LUs that are separated from the rest of the nursing home. Considering this result, the architectural separation may be an important factor for features of specialised LUs for dementia, such as homelike meal provision.

6. Resident-per-staff member ratios are lower in small than in large scale LUs, but the number of LUs without nursing staff during the assessed time periods was higher in small LUs.

Small and large LUs differ regarding their staff ratios and organisation. Small LUs had a better staff ratio, but registered nurses were not always present in small LUs and were not constantly assigned to the LU in small LUs. We assume that it is difficult for nursing homes to realise such low resident-per-registered nurse ratios, and nursing homes have to assign registered nurses to other LUs in the nursing homes. To our knowledge, staff ratios and assignment was not the focus of studies on the effect small LUs until now, so the influence of these factors remains unclear; studies on staff ratios in large specialised LUs for PwD indicate their relevance (see next point).

7. A high percentage of head nurses with a special qualification for psychogeriatric care and a lower ratio of residents-per-registered nurse is a distinctive feature of large segregated LUs with additional funding.

The difference regarding the staffing of large segregated LUs with additional funding and the other large LUs can be explained by the legal agreement of criteria that define the special services in those LUs. Amongst other aspects, the qualification of the head nurse and better staff ratios are mandatory. As it is reported for other specialised LUs for dementia [13], it stands to reason that the residents from this LU are selected based on admission criteria. In our sample, in these LUs were living more residents with severe dementia, what is associated with increased requirements of providing a good quality of care and an increased psychological distress of carers [33]. Segregated LUs, which receive additional funding, have the possibility to and actually provide more and higher educated staff to account for the residents’ structure.

Current reviews found a positive relationship between nursing staff ratios resp. qualification and some indicators of quality of care [34, 35] but not for the quality of life of the residents [36]. Studies that focus explicitly on residents with dementia are sparse. Two studies have investigated the association between staffing levels resp. qualification and dementia-specific outcomes in assisted living facilities and in nursing homes [37] and in dementia special care units [38]. Zimmerman and colleagues [37] found a positive but small influence of a specialised worker approach on residents’ with dementia quality of life but not of nursing staff ratios. In the study from Abrahamson et al. [38], nursing assistant staff ratios were a predictor of residents’ with dementia quality of life; registered nurse-to-resident ratios seemed to have no influence. These results from the literature and our findings demonstrate that staff ratios need to be considered in LU evaluation studies, especially in comparing LUs with differing staff ratios. However, regarding the calculation of staff ratios in nursing homes, a consensus on the most valid measure does not exist [39] and studies use different approaches in investigating staff, which makes comparisons of findings difficult [40]. Staff ratios like they are used in this or the cited studies ignore local conditions, such as resident case mix and time spent on indirect care, such as administrative work. Therefore, staff ratios can solely be interpreted as an indicator that notes a relevant aspect that requires further attention.

### Limitations

There are methodological constraints of the study De-menzMonitor and the present investigation that limit the external validity of the results. The study DemenzMonitor is conceptualised as an explorative study, which means that no power analysis was calculated to detect and validate suspected differences between the groups. The sample size in the groups was different and was small for small LUs. Because of the sampling method of the study, the results are potentially subject to a selection bias. Therefore, the results cannot be seen as representative of German nursing homes in general. The average number of places of the participating nursing homes is higher than those reported in the national care statistics [41]. Possibly, the environmental characteristics differ in smaller nursing homes. As reported, the LUs are located in nursing homes with or without a specialisation, but the specialisation of the nursing home was not part of this analysis. We do not know if some of the characteristics also depend on the concept or the organisation of the nursing home.

The content validity of the questionnaire was tested in expert groups and cognitive interviews; nevertheless, misunderstandings in terms and definitions are not always avoidable. This may have influence on the reliability and validity of the findings. However, reliability testing has not been performed yet. Therefore, the validity of the results has to be interpreted with caution.

Regarding the calculation of staff ratios, time periods as they were used in this study give a constraint insight. Although staff ratios were quite constant in our investigation during four different time periods during the day, staff fluctuation may cause a change of these ratios. Therefore, staff ratios that are calculated in defined time periods have to be interpreted as snapshots and don’t allow an estimation of the situation during the whole day.

In this investigation, the definition of LUs was based on the following characteristics: living concept, size and funding. The LUs were allocated to the defined types based on these indicators and not based on an item assessing the concept of the LU. This approach could have caused a wrong allocation, because of the varying understanding of the concepts for specialised LUs.

## Conclusion

The description of the defined LU types showed that there are considerable differences between LUs that depend either on the living concept or the size. Some characteristics do not differ according to the living concept ‘segregated vs. integrated’, respectively ‘small or large’. Our assumption that large segregated LUs provide more specialised registered nurses only applied to LUs with additional funding. In our sample, better staff ratios and qualifications were not characteristics of large segregated LUs in general. Although both types host considerably more residents with severe dementia than others, only LUs with additional funding engaged more and higher educated staff. In Germany, the policy questions, if specialised LUs should receive additional funding and governments should regulate the expenditure of this money, are not answered finally. Therefore, we suggest an investigation of the association between staff ratios as a characteristic of specialised LU with and without additional funding and quality of care and quality of life of residents with dementia.

In small LUs, we only found expected homelike features (such as homelike meal provision) in the integrated LUs. Small segregated LUs did not differ from other LUs with regard to homelike features. This finding highlights the difficulty of implementing these features in complex organisations such as nursing homes. If the effect of homelike features is evaluated, the features should be defined clearly, and studies should verify that LUs actually implement them.

Future studies that evaluate specialised LUs should consider a definition, which takes the discussed characteristics into account. A definition that is solely based on the size or the living concept ignores the diversity within these groups. Clear descriptions of LU characteristics are always needed in evaluation studies; otherwise, the influence of structural differences cannot be estimated.

The structure of a LU can facilitate quality of care and quality of life of residents, but it may also be an empty shell, if the content of care and underlying interpersonal relationships do not address the resident’s needs. Existing concepts of specialised LUs aim to improve clinical as well as psychosocial outcomes to achieve an improvement of quality of care and quality of life; nevertheless the existing concepts can be discussed critically. When specialised LUs are conceptualised as segregated LUs - even as locked ones - residents may have better clinical outcomes because of better staff ratios and qualification, but at the same time, segregated (locked) units provoke separation and may not support the idea of participatory activities beyond the unit and the nursing home. From the societal perspective, it must be questioned if segregated concepts undermine the idea of social inclusion, which is determined as a human right by the United Nations [42]. For the further development of concepts for LUs a critical discussion is obligatory, which structure is necessary to implement person-centred concepts of care and enhance quality of life of the residents.
